# Development of polysaccharide-coated layered double hydroxide nanocomposites for enhanced oral insulin delivery

**DOI:** 10.1007/s13346-023-01504-7

**Published:** 2024-01-12

**Authors:** Huiwen Pang, Youzhi Wu, Yang Chen, Chen Chen, Xuqiang Nie, Peng Li, Guojun Huang, Zhi Ping Xu, Felicity Y. Han

**Affiliations:** 1 Australian Institute for Bioengineering and Nanotechnology, The University of Queensland, Brisbane, QLD 4072 Australia; 2 School of Biomedical Sciences, Faculty of Medicine, The University of Queensland, Brisbane, QLD 4072 Australia; 3 College of Pharmacy, Zunyi Medical University, Zunyi, 563006 China; 4 Key Lab of the Basic Pharmacology of the Ministry of Education & Joint International Research Laboratory of Ethnomedicine of Ministry of Education, Zunyi Medical University, Zunyi, 563006 China; 5 Hainan Beautech Stem Cell Anti-Aging Hospital, Hainan, 571400 China

**Keywords:** Oral insulin, Layered double hydroxide (LDH), Chitosan, Alginate, Hypoglycemic effect

## Abstract

Oral insulin (INS) is predicted to have the most therapeutic advantages in treating diabetes to repress hepatic glucose production through its potential to mimic the endogenous insulin pathway. Many oral insulin delivery systems have been investigated. Layered double hydroxide (LDH) as an inorganic material has been widely used in drug delivery thanks to its appealing features such as good biocompatibility, low toxicity, and excellent loading capability. However, when used in oral drug delivery, the effectiveness of LDH is limited due to the acidic degradation in the stomach. In this study, to overcome these challenges, chitosan (Chi) and alginate (Alg) dual-coated LDH nanocomposites with the loading of insulin (Alg-Chi-LDH@INS) were developed by the layered-by-layered method for oral insulin delivery with dynamic size of ~ 350.8 nm, negative charge of ~  − 13.0 mV, and dispersity index 0.228. The insulin release profile was evaluated by ultraviolet–visible spectroscopy. The drug release profiles evidenced that alginate and chitosan coating partially protect insulin release from a burst release in acidic conditions. The analysis using flow cytometry showed that chitosan coating significantly enhanced the uptake of LDH@INS by Caco-2 cells compared to unmodified LDH and free insulin. Further in the in vivo study in streptozocin-induced diabetic mice, a significant hypoglycemic effect was maintained following oral administration with great biocompatibility (~ 50% blood glucose level reduction at 4 h). This research has thus provided a potential nanocomposite system for oral delivery of insulin.

**Graphical Abstract:**

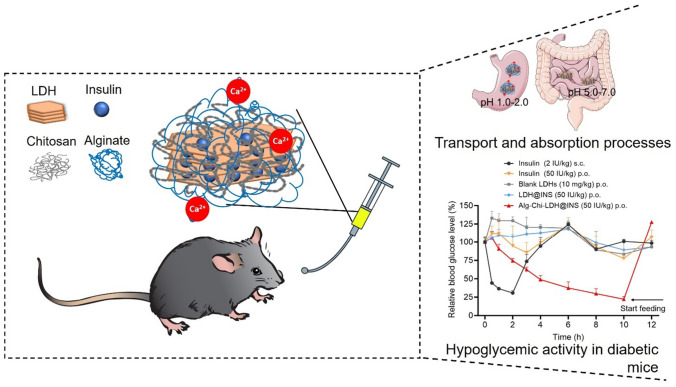

**Supplementary Information:**

The online version contains supplementary material available at 10.1007/s13346-023-01504-7.

## Introduction

Currently, relying on exogenous insulin (INS) is the only practical way to control blood glucose levels for patients with late-stage type 2 diabetes or type 1 diabetes [1]. Compared to traditional subcutaneous injections of insulin, oral insulin is much more convenient and may largely improve patients’ compliance [2]. Oral insulin mimics the normal insulin absorption pathway to establish a portal-peripheral insulin gradient, which avoids peripheral hyperinsulinemia raised by frequent subcutaneous injections [3]. However, for oral insulin to exert its hypoglycemic effects, its delivery must overcome biochemical and physical barriers, including gastric acid and gastrointestinal enzymes, as well as the mucosal and epithelial cell layers of the intestines [4]. Oral insulin absorbed through the portal vein is then transported to the liver, where it rapidly reduces blood glucose levels [5]. Therefore, many formulation methods have been examined for oral insulin delivery to address these challenges, such as liposome [6] and PLGA [7]. However, to date, no oral insulin product is available in the market.

Layered double hydroxides (LDHs) are specific lamellar materials consisting of octahedral sheets with metallic cations in the center [8]. LDHs possess desirable properties that include the ability to customize layer charge density and particle size, strong biocompatibility, and minimal toxicity. LDHs have been widely utilized in drug delivery for the treatment of various diseases, including cancers and cardiovascular diseases, due to their unique properties [9, 10]. These properties include full protection of intercalated drugs by the LDH metal hydroxide layers, low cytotoxicity, and good biocompatibility to mammalian cells, and the capability for “endosome escape,” resulting in a burst release of LDHs from endosomal compartments [11, 12]. Besides, compared to liposome or PLGA et al. organic materials, LDHs hold lower cost, more facile preparation, and convenient drug loading [13]. However, it is important to note that LDHs have a natural chemical structure that makes them susceptible to rapid degradation under acidic conditions [14]. Therefore, coating with chitosan and alginate may be a potentially efficient way against acidic degradation owing to the merits of the two natural polysaccharides. Chitosan (Chi) has been demonstrated to enhance drug absorption by virtue of its mucoadhesive properties and its ability to disrupt tight junctions between epithelial cells. This facilitates the transport of macromolecular drugs through the paracellular pathway [15]. Alginate (Alg) is a highly suitable natural polymer for blending with chitosan due to its anionic nature, which complements the cationic backbone of chitosan, resulting in the formation of a more stable nanomaterial. Alginate typically undergoes shrinkage at low pH and dissolution at high pH values. On the other hand, chitosan dissolves at low pH levels but becomes insoluble at high pH values [16]. Thus, creating polyelectrolyte complexes between these two materials has become of important research interest for overcoming the limitations of each material [16].

In this study, an LDH-based nanocomposite was developed to achieve effective oral delivery of insulin. The LDH was synthesized to associate with insulin via electrostatic interactions and hydrogen bonding (LDH@INS), which effectively protected the insulin from degradation in the physiological environment; then the alginate and chitosan-coated LDH@INS (Alg-Chi-LDH@INS) nanocomposites were prepared by layer-by-layer coating method. In vitro cell cytotoxicity and cell uptake of Chi-LDH@INS were evaluated in Caco-2 cells. Finally, the hypoglycemic effects of the doubly polysaccharide-coated LDH@INS nanocomposites were assessed in streptozocin-induced diabetic mice.

## Materials and methods

### Materials

Ferric chloride (FeCl_3_), magnesium chloride (MgCl_2_), sodium hydroxide (NaOH), insulin (human recombinant, 91077C), fluorescein isothiocyanate isomer I (FITC, F7250), streptozocin (S0130), acetic acid (695092), chitosan (448869, Mw 50,000–190,000 Da), Fluoroshield™ with DAPI (F6057), BCA protein assay kit (BCA1-1KT), and calcium chloride (CaCl_2_) were purchased from Merck (St. Louis, MO, USA). Sodium alginate (E410, NaC_6_H_7_O_6_, Mw: 10,000–600,000; M/G ratio: 1:1) was purchased from the Melbourne Food Depot. These chemicals were used without purification. Phosphate-buffered saline (PBS, 10010023) buffer and Dulbecco’s modified eagle medium (DMEM, 11965118) were obtained from Life Technologies Corporation (Mulgrave, VIC, Australia). Fetal bovine serum (FBS, 10099141) was bought from Gibco (St. Louis, MO, USA). Centrifuge 5415R (Eppendorf) was used in the research. The ultrapure water used throughout all experiments was purified using a Milli-Q Plus 85 (Millipore, Bedford, MA, USA).

### Synthesis of LDH and LDH@INS

MgFe-LDH was prepared via the co-precipitation method modified from the reported work [17]. An aqueous solution containing 0.15 M MgCl_2_ (Sigma-Aldrich, 208337) and 0.05 M FeCl_3_ (Sigma-Aldrich, 157740) and an aqueous solution containing 0.4 M NaOH (Sigma-Aldrich, S5881) were mixed at the equal volume and vigorously stirred for 1 h in an ice bath. The precipitate was collected by centrifugation (13,000 rpm, 10 min) and washed twice with deionized water and resuspended in deionized water. The concentration of LDHs was calculated based on the dry weight of LDH. Specifically, a certain volume of LDH in deionized water was dried in an oven at 80 °C for 1 day. Dividing the mass of completely dry LDH by the volume gave the concentration of LDH. For preparing LDH@INS suspension, a certain amount of insulin in 1 ml (0.1–2.0 mg/ml insulin) was mixed with 1 ml of LDH suspension (1.0 mg/ml) and stirred for 1 h (600 rpm). Then, the supernatant was collected by centrifuging and the free insulin concentration was measured using BCA kit at 562 nm by UV–Vis (Fig. S1). The insulin loading capacity and efficiency was calculated using the following equation:

$$Loading\;capacity\;(\%)\:=\:(total\;amount\;of\;insulin\:-\:unencapsulated\;insulin)\;/\;total\;final\;weight\;of\;the\;LDH@INS\:\times\:100\%$$

$$Loading\;efficiency\;(\%)\:=\:(total\;amount\;of\;insulin\:-\:unencapsulated\;insulin)\;/\;total\;amount\;of\;insulin\:\times\:100\%$$

To gain the insight of adsorption mechanism of insulin on LDHs, the Langmuir model (*Qe* = *QmCe*/(1/*K* + *Ce*); *Ce*: equilibration concentration of insulin in solution (mg/ml); *Qe*: equilibration amount of insulin adsorbed by LDH (mg/mg); *Qm*: the maximum monolayer adsorption amount (mg/mg); *K*: adsorption equilibrium constant (ml/mg)) was applied to fit the adsorption isotherm.

### Synthesis of polysaccharide-coated LDH@INS nanocomposites

**Scheme 1:**
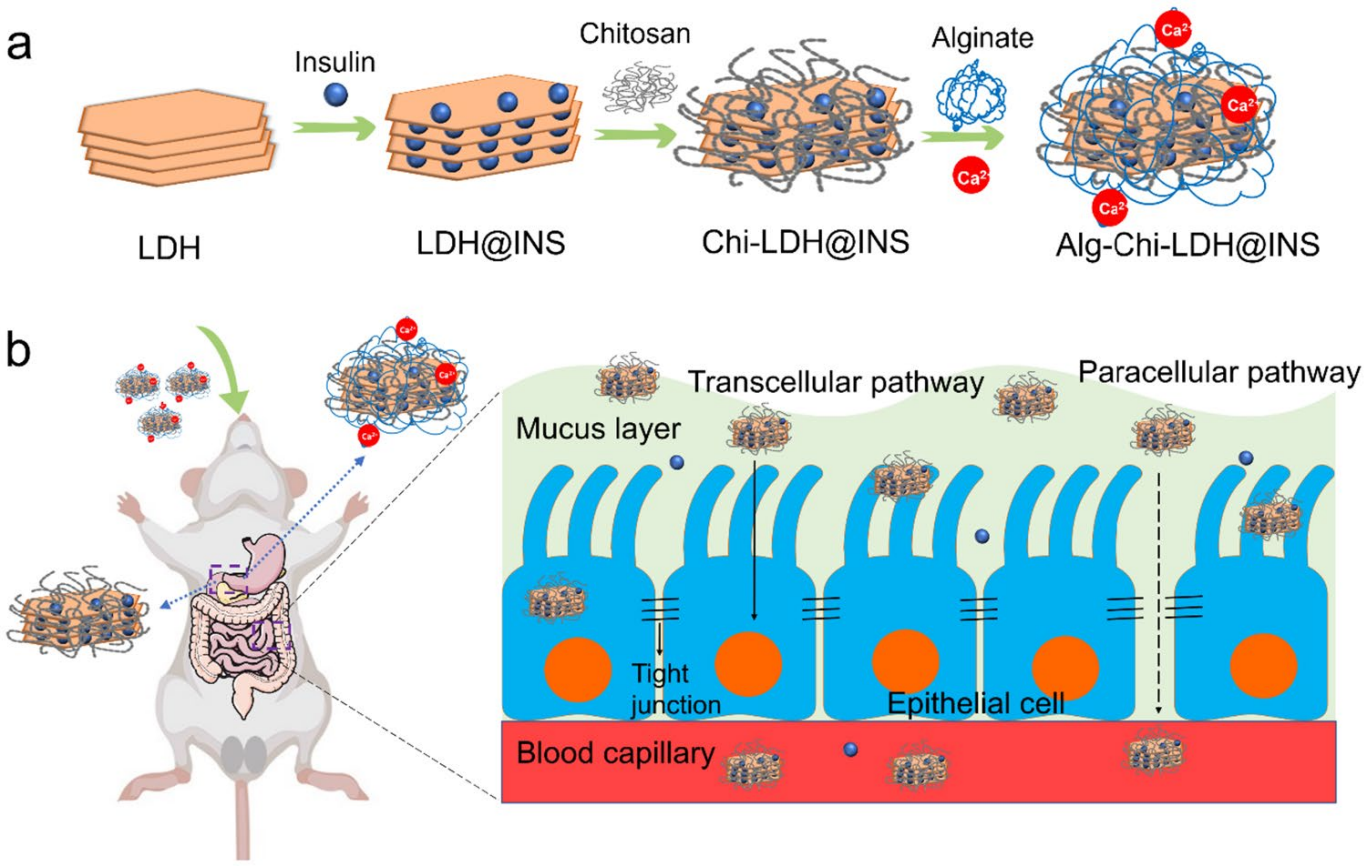
Schematic diagram of the synthetic process (**a**) of Alg-Chi-LDH@INS nanocomposites and the oral delivery of insulin (**b**) through LDH-based nanocomposites for diabetic treatment. The paracellular pathway, indicated by dashed line, is predicted to be achieved in this study

Alginate and chitosan-coated LDH@INS nanocomposites were prepared via a layer-by-layer approach [6, 17], as shown in Scheme 1. Briefly, 200 μl of chitosan solution (0.5%, wt/v) in 1% acetic acid (pH 5) was dropwise added into 1 ml LDH@INS suspension (1 mg LDH) under stirring. After stirring for 45 min at 600 rpm, the nanocomposites were collected and washed with deionized water via centrifugation (13,000 rpm, 10 min) to get the Chi-LDH@INS nanocomposites. To make Alg-Chi-LDH@INS, 200 μl alginate solution (0.5%, wt/v) was added into 1 ml Chi-LDH@INS suspension (1 mg LDH) under stirring at 600 rpm. After stirring for 45 min, the pellets were collected and wash with deionized water via centrifuging (13,000 rpm, 10 min). Finally, 50 μl of 5.0 M CaCl_2_ solution was added into the suspension (1 ml Alg-Chi-LDH@INS, 1 mg LDH) to crosslink alginate to get Alg-Chi-LDH@INS nanocomposites, which were finally collected via centrifugation (13,000 rpm, 10 min). The LDH-based nanocomposites were fresh prepared and stored at 4 °C for cell and animal experiments.

### Characterization of the Alg-Chi-LDH@INS nanocomposites

The hydrodynamic particle size and charge of the sample nanocomposites were determined by dynamic light scattering (DLS) using DTS1070 folded capillary cell (Malvern) on a Malvern Zetasizer at the room temperature. Transmission electron microscopy (TEM) images were captured in HITACHI HT7700 after the sample suspension was dropped and dried on the Cu grid. Fourier transformed infrared (FTIR) spectra were performed on a Nicolet 6700 FTIR spectrometer in the range of 400–4000 cm^−1^ at the resolution of 2 cm^−1^ with 32 scans.

### The stability of Alg-Chi-LDH@INS in various stimulated biological fluids

To understand the colloid stability of Alg-Chi-LDH@INS for oral insulin delivery, the particle size distribution of Alg-Chi-LDH@INS in various simulated biological fluids was measured using DLS. Briefly, 1 ml of Alg-Chi-LDH@INS (initial LDH 1 mg) nanocomposites was mixed with 10 ml of the simulated gastric fluid (pH 1.2), the simulated intestinal fluid (SIF, pH 6.8), and PBS (pH 7.4), respectively, and incubated for 0.5–2 h at 37 °C. The particle size and distribution of the samples were determined, and the morphology was observed by the TEM (HITACHI HT7700).

### Insulin release in vitro

In brief, LDH@INS and Alg-Chi-LDH@INS (initial LDH 1 mg) were mixed with 1 ml of the simulated gastric fluid at pH 1.5 and incubated for 2 h at 37 °C, respectively. At certain time point, 100 μl of the solution was taken from mixture solution and replaced with the same volume of fresh buffer solution. The concentration of insulin released from these nanocomposites was examined by BCA kit on a UV–Vis spectrometer at 562 nm. After 2-h incubation in simulated gastric fluid, these nanocomposites were collected and transferred into the simulated intestinal fluid at pH 6.8 for another 2 h and finally transferred into PBS (pH 7.4) for 2 h. The released insulin from the nanocomposites was monitored using the same process as in the simulated gastric fluid.

### Cytotoxicity in Caco-2 cell line

The MTT (3-(4,5-dimethylthiazol-2-yl)-2,5-diphenyltetrazolium bromide, M6494, Thermo Fisher Scientific) assay was used to evaluate the cytotoxicity of Chi-LDH@INS. To measure the cells viability, the Caco-2 cells were seeded in 96-well plates at the cell density at a 1 × 10^4^ per well in 100 μl of DMEM with 10% FBS at 37 °C in 5% CO_2_ incubator. After 24 h incubation, the medium was removed and 100 μl insulin-loaded Chi-LDH@INS with the concentrations of 0, 10, 100, 500, and 1000 μg/ml and blank solution was added to that well for 24 h, respectively. Afterwards, 20 μl of MTT (5 mg/ml in PBS solution) solution was dropwise added to each well and the cells were then incubated at 37 °C in 5% CO_2_ for 4 h. The medium was removed, and 150 μl of dimethyl sulfoxide (DMSO) was then added into each well with 10 min of low-speed oscillation and tested using a microplate reader at 490 nm. Experiments were executed five times.

### Cellular uptake by Caco-2 cell line

To test the cell uptake of coated and uncoated LDHs, Caco-2 cells were seeded at 2 × 10^5^ cells per well in 6-well plates and incubated at 37 °C for 24 h. Fluorescein isothiocyanate (FTIC) was conjugated to insulin, according to the previous study [18]. The cells were then separately incubated for 5 h with FITC-insulin, LDH@FITC-INS, or Chi-LDH@FITC-INS nanocomposites. Then, the culture medium was removed, and the cells were washed 3 times with PBS. Next, cells were digested using 0.25% trypsin and were centrifuged, collected, dispersed, and finally renewed in a solution of 0.2 ml PBS for detection by flow cytometry. Caco-2 cells were also co-incubated with Chi-LDH@FITC-INS nanocomposites for 1, 3, and 5 h and then incubated with 4% paraformaldehyde at 25 °C for 20 min. After rinsing three times with PBS, the cover slips with cells were coved on the slide with one drop of Fluoroshield™ with DAPI. A Leica TCS SP8 MP confocal microscope (Leica Microsystems, Mannheim, Germany) was used to conduct confocal laser scanning microscopy (CLSM).

### In vivo assessment in mice

Animal experiments were performed in accordance with approval from the University of Queensland Animal Ethics Committee, Australia (the approval number was 2022/AE000346, and the date of approval was 01 Jan 2023) and were conducted in accordance with the National Health and Medical Research Council of Australia’s Australian Code of Practice for the Care and Use of Animals for Scientific Purposes (8th Edition, 2013). C57BL/6 J mice (male, 8 weeks old) were purchased from the Animal Resources Centre, Western Australia. All animals were housed within the University of Queensland Biological Resources animal facility in a pathogen-free environment with 12 h/12 h dark/light cycle and free access to food and water.

#### Biocompatibility in health mice

Animals were randomly divided into two groups (*n* = 5). Experimental group received daily oral dose of LDH nanocomposites (10 mg/kg, without insulin loaded) for 28 days; the other group received oral administration with the equal volume of PBS for 28 days.

Body weights were recorded every 4 days. After 28 days treatment, whole blood was collected from tail for hematology analysis by the Mindray BC-5000 Vet (Shenzhen, China). Then, the mice were sacrificed, and hearts, livers, spleens, lungs, kidneys, testes, and intestines were taken out, fixed by 4% paraformaldehyde, and stained by hematoxylin and eosin (H&E).

#### Hypoglycemic activity in diabetic mice

Diabetic mice were induced by intraperitoneal injection of streptozotocin (STZ) at a single dose of 180 mg/kg. After 1 week, the mice with fasting blood glucose level (BGL) over 13.88 mM standard were selected for further experiment [19].

The diabetic mice were fasted overnight and divided into five groups (*n* = 5) and received the following formulas: insulin solution (2 IU/kg, subcutaneous injection (s.c.)), insulin solution (50 IU/kg, oral (p.o.)), blank LDHs (10 mg/kg, p.o.), LDH@INS nanocomposites (50 IU/kg, p.o.), Alg-Chi-LDH@INS nanocomposites (50 IU/kg, p.o.). At designed time point, the BGL was measured by a Statstrip glucose meter (Nova Biomedical). Relative blood glucose level (%) was calculated using the following equation:

$$Relative\;blood\;glucose\;level\;(\%)\:=\:Blood\;glucose\;level\;(mM)\;/\;Initial\;blood\;glucose\;level\;(mM)\:\times\:100\%$$

### Statistic analysis

All data are expressed as the mean ± standard deviation (SD). Statistical analyses were performed using GraphPad Prism™ v9.4.1 (GraphPad Software, La Jolla, USA). Significant levels were determined using one-way ANOVA followed by Dunnett’s test. The *P* values less than 0.05 were statistically significant.

## Results and discussion

### Characterization of insulin-loaded LDHs with alginate-chitosan coating

**Fig. 1:**
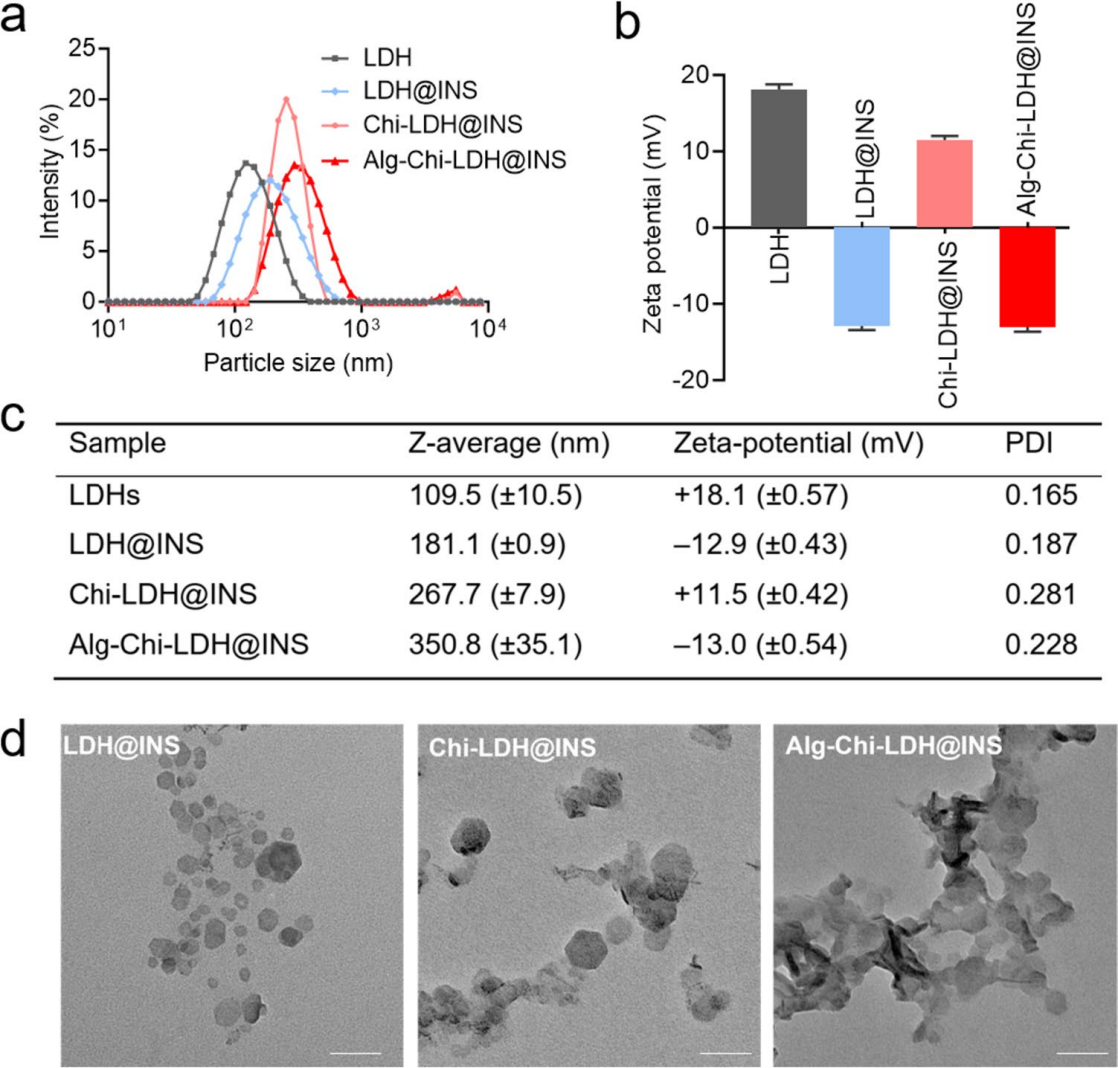
Characterization of insulin (INS)-loaded LDH-based nanocomposites. The size distribution (**a**), zeta potentials (**b**), dispersity index (PDI) (**c**), and transmission electron microscopy images, scale bar = 500 nm, *n* = 3 (different samples of the same batch) (**d**) of LDH@INS, Chi-LDH@INS, and Alg-Chi-LDH@INS nanocomposites

**Fig. 2:**
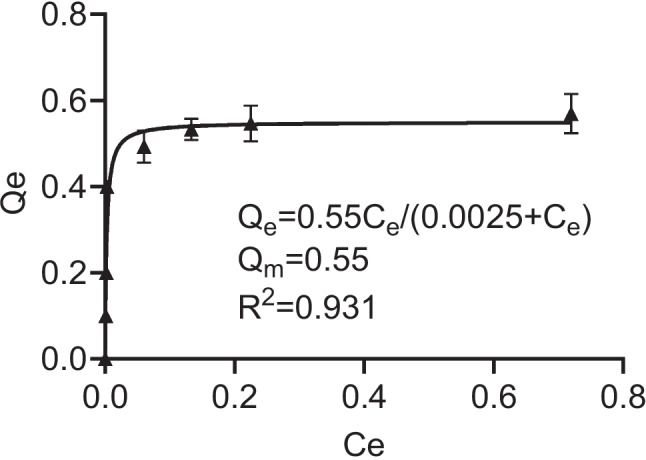
Insulin loading profile on LDHs. Adsorption isotherm of insulin on LDHs fitted in Langmuir model with *R*.^2^ being 0.931 and maximum monolayer adsorption capacity (*Qm*) being 0.55 mg insulin per mg LDH. The data are expressed as mean (± SD). *Ce*: equilibrium concentration of adsorption of insulin (mg/ml), *Qe*: equilibrium amount of adsorbed insulin (mg/mg LDH), *n* = 3 (different samples of the same batch)

Insulin-loaded LDHs with coating of chitosan and alginate were prepared using a layer-by-layer method [6, 17], as shown in Scheme 1. LDHs had the average hydrodynamic size around 109.5 nm and the zeta potential of + 18.1 mV (Fig. 1). The morphology of the LDHs had hexagonal shapes and the size was smaller in TEM images than that measured by DLS, which is likely due to the slight aggregation. The insulin loading profile to LDHs was evaluated. With increasing amounts of insulin added into LDHs, the zeta potential of the LDHs gradually decreased from positive to negative (Fig. S2). The loading efficiency of insulin was well fitted with Langmuir adsorption model (*R*^2^ = 0.931) (Fig. 2), suggesting the monolayer adsorption of insulin on LDHs. Based on the Langmuir model, the maximal insulin loading capacity was 0.55 mg/mg and the adsorbed insulin was mutually independent and tied in a single layer with the adsorbent surface. The monolayer adsorption processes were also reported in many other drugs to LDHs, such as BSA [20] and Congo red [21]. To ensure the maximum drug loading of insulin, a 0.6 mg insulin solution in 1 ml was added to a 1 mg LDH solution in 1 ml with the loading capacity of 35.48% and loading efficiency of 91.67%.

After loading insulin on LDHs, the hydrodynamic size of LDH@INS slightly increased from 109.5 to 181.1 nm and its zeta potential changed from + 18.1 to − 12.9 mV. The hydrodynamic size of Chi-LDH@INS increased to 267.7 nm after coating with chitosan, and the nanocomposites became positively charged with the zeta potential of + 11.5 mV. With the further coating of alginate on Chi-LDH@INS, the size of Alg-Chi-LDH@INS further increased to 350.8 nm and the nanocomposites turned negatively charged with the zeta potential of − 13.0 mV. The constant increased particle sizes and alternated zeta potential changes on LDHs suggest that chitosan and alginate were successfully coated on LDH@INS nanocomposites. The morphology of the final formulation kept hexagonal. However, compared to LDH@INS, after the further coating of chitosan or/and alginate, the formulation exhibited stronger aggregation (Fig. 1d), which also may explain the increased size of the DLS results.

Besides, according to the FTIR spectra analysis, as shown in Figure S3, after absorbing insulin on LDHs, a few new bands at 1642 and 1510 cm^−1^ were observed for LDH@INS nanoparticles due to C = O stretching vibration and N–H bending vibration [22]. Furthermore, one of the most characteristic bands from chitosan and alginate located at 1025 cm^−1^ (CH_2_–OH in primary alcohol [23]) was observed after further coating of alginate and chitosan, which further demonstrated the successful formulation of Alg-Chi-LDH@INS.

### The insulin release profiles of Alg-Chi-LDH@INS

**Fig. 3:**
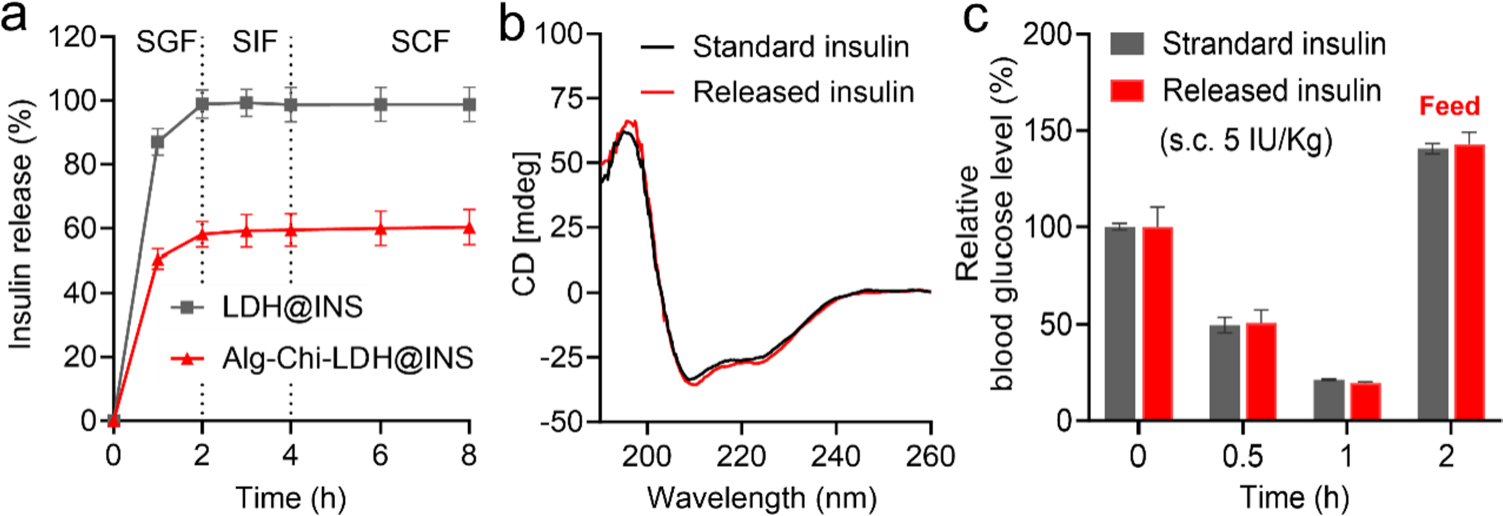
In vitro insulin release profiles and bioactivate test of released insulin in vitro and in vivo. Insulin in vitro release profile from LDH@INS and Alg-Chi-LDH@INS nanocomposites in simulated gastric fluid (pH 1.2, 2 h), simulated intestinal fluid (pH 6.8, 2 h), and PBS buffer (pH 7.4, 2 h) (**a**); the circular dichroism spectra (**b**) of the released insulin from Alg-Chi-LDH@INS; hypoglycemic effects (**c**) of the released insulin from Alg-Chi-LDH@INS in vivo in STZ-induced diabetic mice via subcutaneous injection (5 IU/kg, *n* = 3, different samples of the same batch)

Insulin release profiles of Alg-Chi-LDH@INS and LDH-INS were examined in various buffers at pH 1.2, 6.8, and 7.4, which simulated stomach fluid, small intestine fluid, and the normal body fluid, respectively. As shown in Fig. 3, approximately 80% of insulin was rapidly released within 30 min, and all the absorbed insulin was fully released from the uncoated LDHs within 2 h at pH 1.2. In strong acid conditions, the rate of reaction is pH dependent, primarily due to the equilibrium between protonated (active) and deprotonated (inactive) hydroxyl groups in LDHs [24]. The hydroxyl groups on the surface of the LDHs undergo protonation, leading to the fast formation of water molecules [25]. As a result, the rapid dissolution of LDHs occurs at low pH and leads to the rapid drug release.

On the contrary, the coated Alg-Chi-LDH@INS exhibited controlled release of insulin in different pH conditions. In acidic conditions (pH 1.2), the alginate and chitosan coating provided partial protection to insulin, resulting in the release of 45% and 58% of the loaded protein within 30 and 120 min, respectively. This protection is attributed to the protonation of carboxyl groups in alginate and the crosslinking between alginate and chitosan [26]. At pH 1.2, the isoelectric point of alginate, the protonation of alginate leads to the formation of dense alginate layers, which effectively shields the LDHs from degradation and inhibits insulin release [27]. As the pH increased to 6.8 and 7.4 (simulating small intestine fluid and normal body fluid), no further release of insulin was observed, indicating that insulin remained bound to the surface of LDHs. This phenomenon can be explained by the strong electrostatic interactions between insulin and LDHs, which prevents further release of insulin [28].

Furthermore, the released insulin from the Alg-GC-LDH@INS was stable in structure (Fig. 3b) and maintained normal biological function (Fig. 3c). These results demonstrated that this functional coating of chitosan and alginate on the LDH surface was significantly beneficial to maintain the bioactivity of insulin and to prevent insulin release in acidic conditions.

### Biocompatibility of the LDH and Chi-LDH nanocomposites

**Fig. 4:**
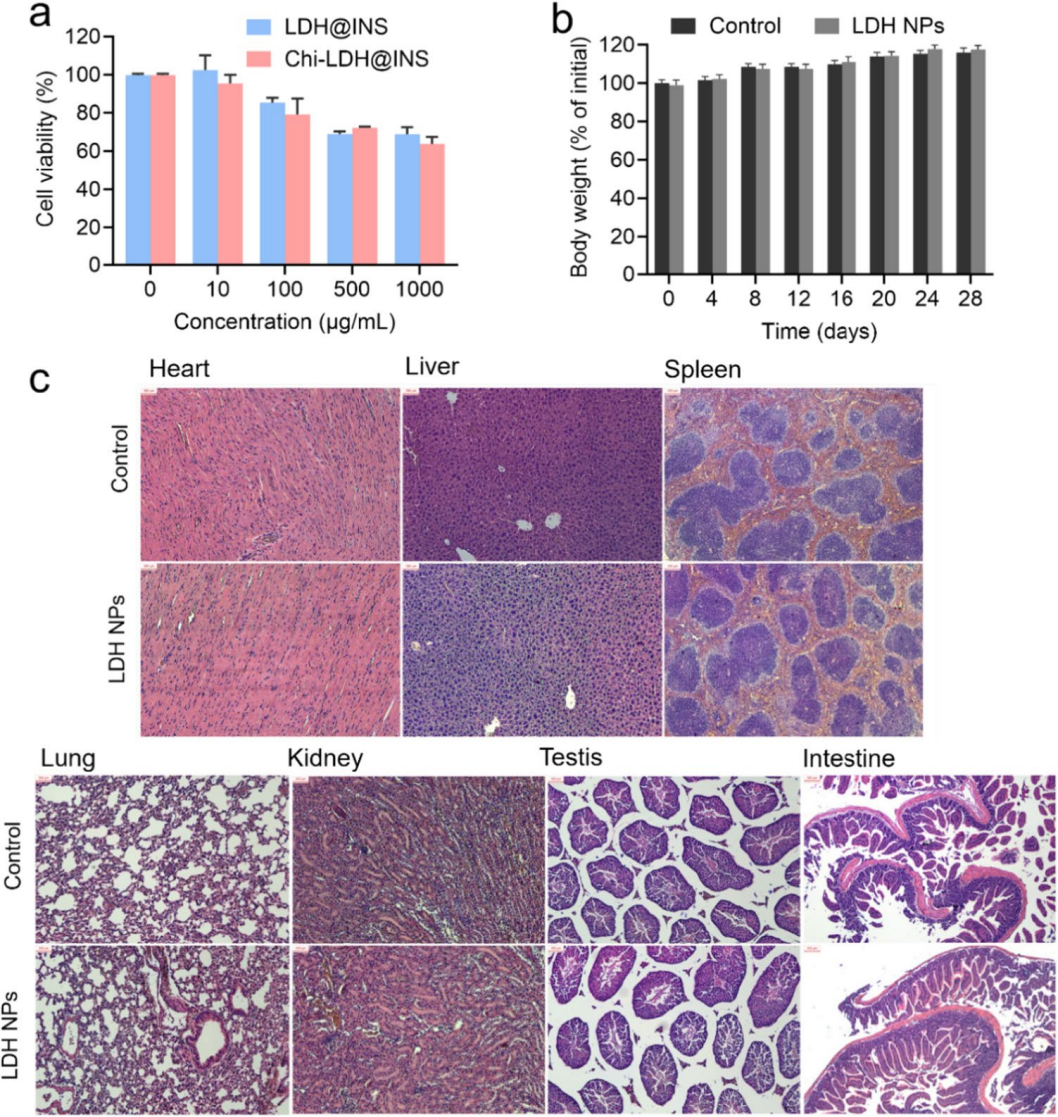
Biocompatibility of the insulin (INS)-loaded LDH-based nanocomposites in vitro with Caco-2 cells (a human intestinal epithelial cell line) (**a**); and in vivo study with mice received LDH nanoparticles orally once a day over 4 weeks compared to PBS solution as control, the changes of (**b)** body weight and (**c)** representative photomicrographs of the heart, liver, spleen, lung, kidney, testis, and small intestine (H&E staining, scale bar = 300 μm, *n* = 5)

In this study, Caco-2 cell line (human colorectal adenocarcinoma cell line), the most commonly used model of intestinal epithelial detection system in vitro [29], was applied to determine the cytotoxicity and cell uptake of LDH nanoparticles due to the fact that cell possess a mucus layer similar with intestinal epithelial cell [29]. The final concentration of the LDH@INS and Chi-LDH@INS nanocomposites in culture medium was from 0 to 1000 μg/ml. As depicted in Fig. 4a, the viability of Caco-2 cells gradually decreased to 60% as the concentration of LDHs and Chi-LDHs increased. However, at concentrations of LDHs or Chi-LDHs equal to or below 100 μg/ml, the cell viability remained around 80%. LDHs have satisfactory biocompatibility within a certain dose range (≤ 100 μg/ml). The similar results were also reported in other cell lines, such as L-132 normal cells [30] and HepG2 cells [31]. It is plausible that LDH nanoparticles initially impede cell proliferation by adhering to the cell membrane, potentially leading to subsequent cell death.

Further to evaluate the biocompatibility of the LDH nanoparticles in vivo, animal body weight and hematology analysis were measured up to 28 days of daily treatment with blank LDHs (10 mg/kg) while control group received only PBS (pH 7.4). Blank LDH, as the principal component of the final formulation (Alg-Chi-LDH@INS), was only applied, considering alginates and chitosan are included in a group of compounds that are generally considered safe by the FDA [32]. Compared to control group, LDH nanoparticles group showed similar healthy hematology results (Figure S4) and weight body gaining (Fig. 4b). No clinical symptoms (fever, diarrhea etc.) and mortality occurred throughout the treatment period. According to the H&E staining (Fig. 4c), there was no significant pathological change in the visceral organs in LDH nanoparticles group compared to control group, which indicated that the tolerable daily intake of LDH less than 10 mg/kg/day (much higher than the total amount of LDH loading 50 IU insulin for one dosage per day) would not raise health risk in vivo. Collectively, these results suggest that LDH nanoparticles exhibited good biocompatibility both in vitro and in vivo. Congruence of such findings was found when LDH was used for ocular drug delivery [33], intramuscular tablets implantation [34], and others [35, 36]. Taken together, these results indicate the good biocompatibility of LDH-based nanocomposites, which is safe to be used in the mouse.

### Cell uptake of the LDH and Chi-LDH nanocomposites by Caco-2 cell line

**Fig. 5:**
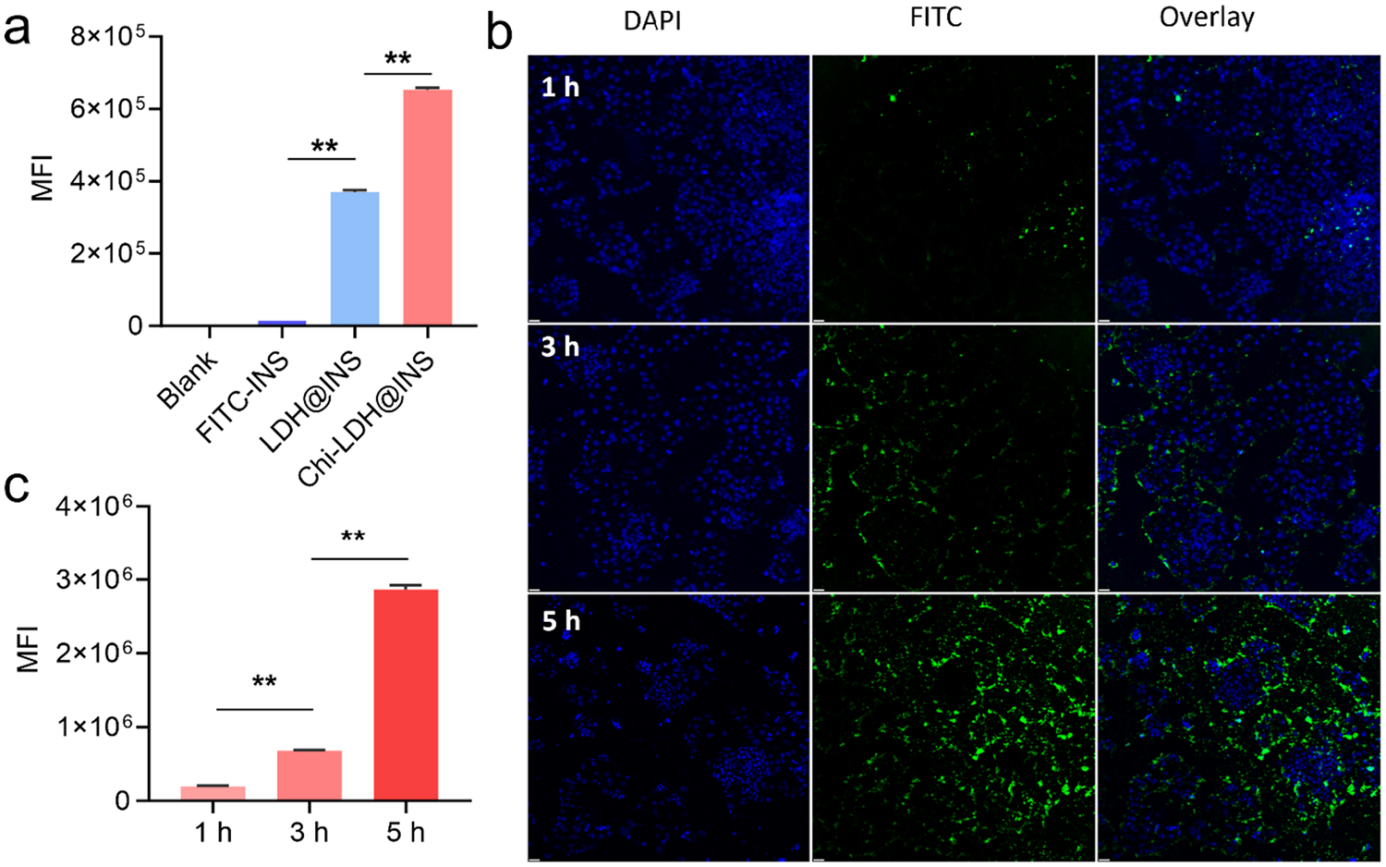
In vitro cellular uptake of the LDH@INS and Chi-LDH@INS nanocomposites by intestinal epithelial cell, Caco-2 cell line. (**a)** Mean fluorescence intensity (MFI) of LDH@FITC-INS and Chi-LDH@FITC-INS after 5-h incubation from flow cytometer, ** means *P* < 0.01; confocal laser scanning microscopy images (**b**) and MFI (**c**) of Chi-LDH@FITC-INS for 1, 3, and 5 h incubation time. Scale bar = 40 μm

To demonstrate the uptake of the LDH-based nanocomposites via the enteral route, Caco-2 cells were used to mimic the uptake in the gastrointestinal tract, in vitro. FITC was conjugated to insulin (Figure S5) and then FITC-labeled insulin was absorbed by the LDH. The cellular uptake of the nanocomposites was examined qualitatively by flow cytometry to assess the internalization by Caco-2 cells. Considering that alginate is an enteric-coated polymer [16], upon reaching the epithelial cells at the intestinal part, it is mostly Chi-LHD@INS. Therefore, LDH@INS and Chi-LDH@INS were determined for cell uptake. As shown in Fig. 5a, the fluorescence intensity of Chi-LDH@INS was significantly higher than that of the control group, free insulin group, and LDH@INS group. Moreover, the fluorescence intensity increased with incubating time of Chi-LDH (Fig. 5b, c), which further indicates the efficient cell internalization of the chitosan-coated LDH nanocomposites. The suggested mechanism for the internalization of chitosan nanocomposites by cells is predominantly through adsorptive endocytosis, which is initiated by nonspecific interactions between the nanocomposites and the cell membranes. This process is mediated, at least in part, by clathrin-mediated processes [37]. Evidence supports that nanoparticles with positively charged surfaces are favorable for epithelial endocytosis, which can be attributed to their cationic nature, enabling better attachment to the anionic cell surface [38]. Besides, abundant saccharide receptors and surface proteins in the Caco-2 cell membrane strongly interact with chitosan, hence promoting the internalization of chitosan-coated LDH@INS nanocomposites into Caco-2 cells [39, 40].

### Hypoglycemic effects in streptozocin-induced diabetic mice

**Fig. 6:**
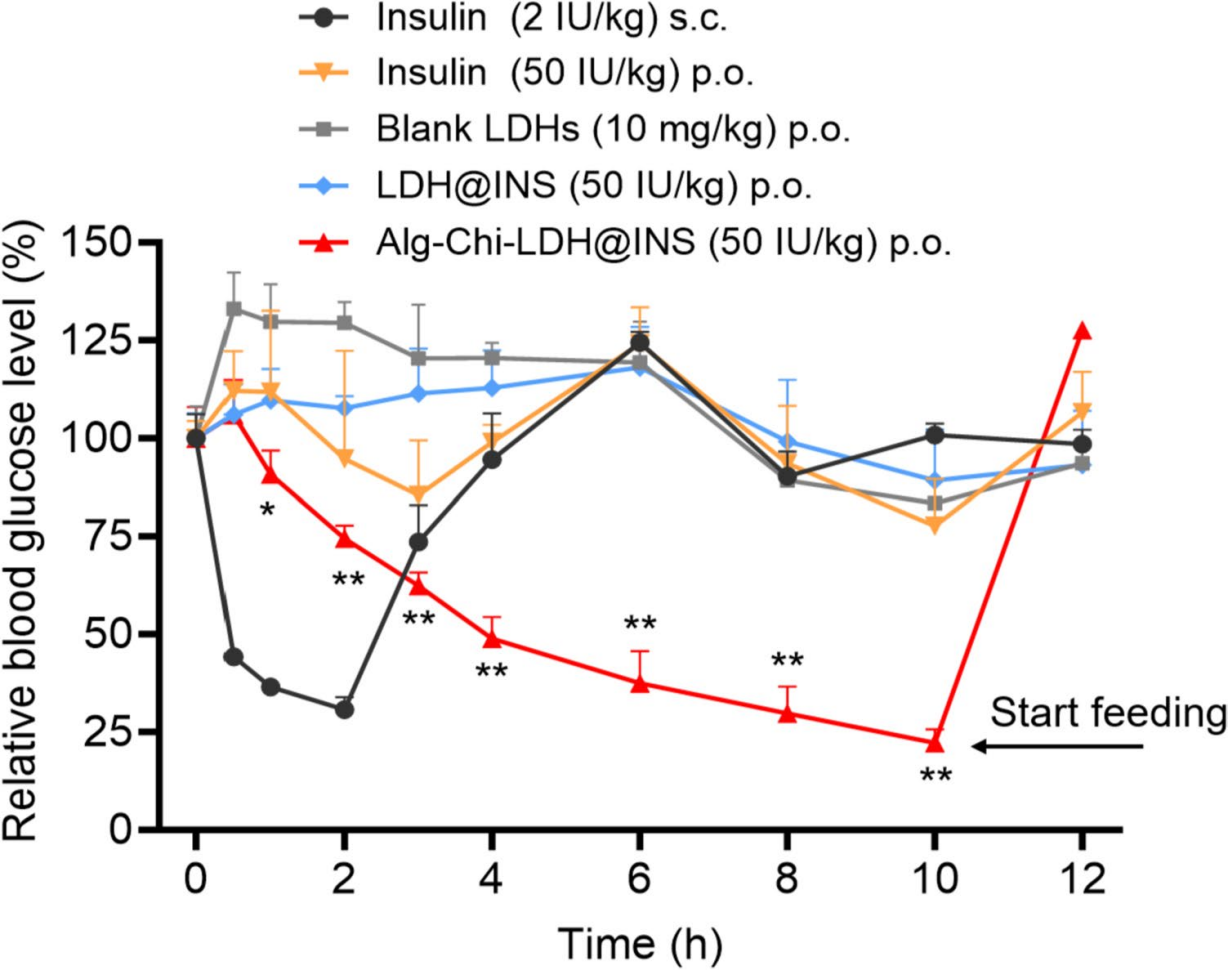
In vivo hypoglycemic effects. Blood glucose levels in diabetic mice after oral (p.o.) administration of different formulations, including blank LDHs (10 mg/kg, p.o.) and free insulin (2 IU/kg, p.o.) as negative control, and LDH@INS (50 IU/kg, p.o.), Alg-Chi-LDH@INS (50 IU/kg, p.o.) as experimental groups; subcutaneous (s.c.) injection of free insulin (2 IU/kg, s.c.) as the positive control (*n* = 5, mean (± SD)). Start feeding: only Alg-Chi-LDH@INS group started feeding at 10 h, others at 12 h. * means *P* < 0.05, ** means *P* < 0.01, compared to LDH@INS group

The effect of hypoglycemic effect from LDH-based nanoparticles was evaluated using streptozocin-induced diabetic mice. As shown in Fig. 6, after 2 h of administration, there were significantly hypoglycemic effects observed with insulin solution (50 IU/kg) or blank LDH nanoparticles solution given orally, while subcutaneous injection of insulin (2 IU/kg) group, as a positive control, sharply reduced the blood glucose level (BGL) and obtained the normal fasting BGL at 2 h. There was no significant reduction of the BGL after oral free insulin directly due to the enzymatic degradation in the gastrointestinal tract and poor membrane permeability [41]. Meanwhile, oral administration of blank LDH group, LDH@INS (50 IU/kg) group, did not decrease the BGL in mice either, which is consistent with the findings reported in another Mg–Al-LDH-based oral insulin formulation [42]. However, there was an increase in the BGL in mice at 4–6 h for blank LDH group. This phenomenon has been previously observed by others and could be attributed to handling and blood sample collection [43]. In contract, encouragingly, about 25%, 50%, and 75% BGL reductions were observed at 2, 4, and 10 h respectively in the group orally administrated with our Alg-Chi-LDH@INS (50 IU/kg) nanocomposites. Considering animal welfare, the food was immediately given to the mice in this group at 10 h. The food for the other groups was provided later at 12 h after the whole test. The administration showed a slower start and longer duration of action, compared to subcutaneous injection of free insulin. Moreover, compared to the insulin-loaded LDH modified with deoxycholic and hyaluronic acids by Huang et al. [42] and other organic biomaterial-based oral insulin formulations, such as PLGA [7], the Alg-Chi-LDH@INS achieved a more significant BGL reduction. The high hypoglycemic efficacy of our designed nanocomposites in the diabetic mice could be attributed to following aspects: (1) The alginate and chitosan-coated nanocarriers serve as an efficient tool for protection of insulin degradation in the physiological environment of stomach; (2) Chitosan coating might enhance the intestine absorption of the insulin drug by increasing the mucoadhesive property, improving cellular uptake and intracellular delivery and paracellular delivery. As shown in Scheme 1, it is predicted that a large amount of insulin was still attached to the LDH form to overcome the mucus and epithelial layer and then into the vein circulation (similar to the in vitro release profile, very limited free insulin being released in pH6.8/7.2 in Fig. 3a).

## Conclusion

In the present study, an insulin-loaded alginate and chitosan-coated LDH nanocomposite system (Alg-Chi-LDH@INS) has been first developed for the oral insulin treatment of diabetes. It was first time to determine that the insulin absorption was well fitted with Langmuir adsorption model, a monolayer adsorption on LDH (0.55 mg insulin/1 mg LDH). The nanocomposite showed a nano scale of hexagon morphology with dynamic size (~ 350.8 nm) and good biocompatibility in a 28-day study in mice. The nanocomposites could protect insulin release in gastric acidic conditions and promoted a high epithelial cell uptake. Oral administration of the Alg-Chi-LDH@INS nanocomposites produced a significant and sustained reduction of hyperglycemia in diabetic mice. Further validation of the Alg-Chi-LDH@INS nanocomposites in the treatment of diabetic patients is warranted. Our initial data have demonstrated that Alg-Chi-LDH nanocomposites have the potential for oral administration of insulin and other unstable macromolecules.

### Supplementary Information

Below is the link to the electronic supplementary material.

Supplementary file1 (DOCX 354 KB)

## Data Availability

The datasets generated during and/or analyzed during the current study are available from the corresponding author upon reasonable request.
