# Supplementary material for: Development of polysaccharide-coated layered double hydroxide nanocomposites for enhanced oral insulin delivery
Source: Drug Deliv Transl Res. 2024 Jan 12;14(9):2345–55. doi: 10.1007/s13346-023-01504-7 (PMC11291568; doi:10.1007/s13346-023-01504-7)
Supplement: Supplementary file 1 — Supplementary file1 (DOCX 354 KB) [file 13346_2023_1504_MOESM1_ESM.docx]

**Supplementary Material**

**Development of polysaccharide-coated layered double hydroxide nanocomposites for enhanced oral insulin delivery**

Huiwen Pang^1^, Youzhi Wu^1^, Yang Chen^2^, Chen Chen^2^, Xuqiang Nie^1,3,4^, Peng Li^1^, Guojun Huang^5^, Zhi Ping Xu^1^*, Felicity Y. Han^1^*

Huiwen Pang^1^, Youzhi Wu^1^, Yang Chen^2^, Chen Chen^2^, Xuqiang Nie^1,3,4^, Peng Li^1^, Guojun Huang^5^, Zhi Ping Xu^1^*, Felicity Y. Han^1^*

^1^ Australian Institute for Bioengineering and Nanotechnology, The University of Queensland, Brisbane, QLD 4072, Australia.

^2^ School of Biomedical Sciences, Faculty of Medicine, The University of Queensland, Brisbane, QLD 4072, Australia

^3^ College of Pharmacy, Zunyi Medical University, Zunyi 563006, China

^4^ Key Lab of the Basic Pharmacology of the Ministry of Education & Joint International Research Laboratory of Ethnomedicine of Ministry of Education, Zunyi Medical University, Zunyi 563006, China

^5^ Hainan Beautech Stem Cell Anti-Aging Hospital, Hainan 571400, China.

* Correspondences: [gordonxu@uq.edu.au](mailto:gordonxu@uq.edu.au) (Z.P.X.); [f.han@uq.edu.au](mailto:f.han@uq.edu.au) (F.Y.H.)


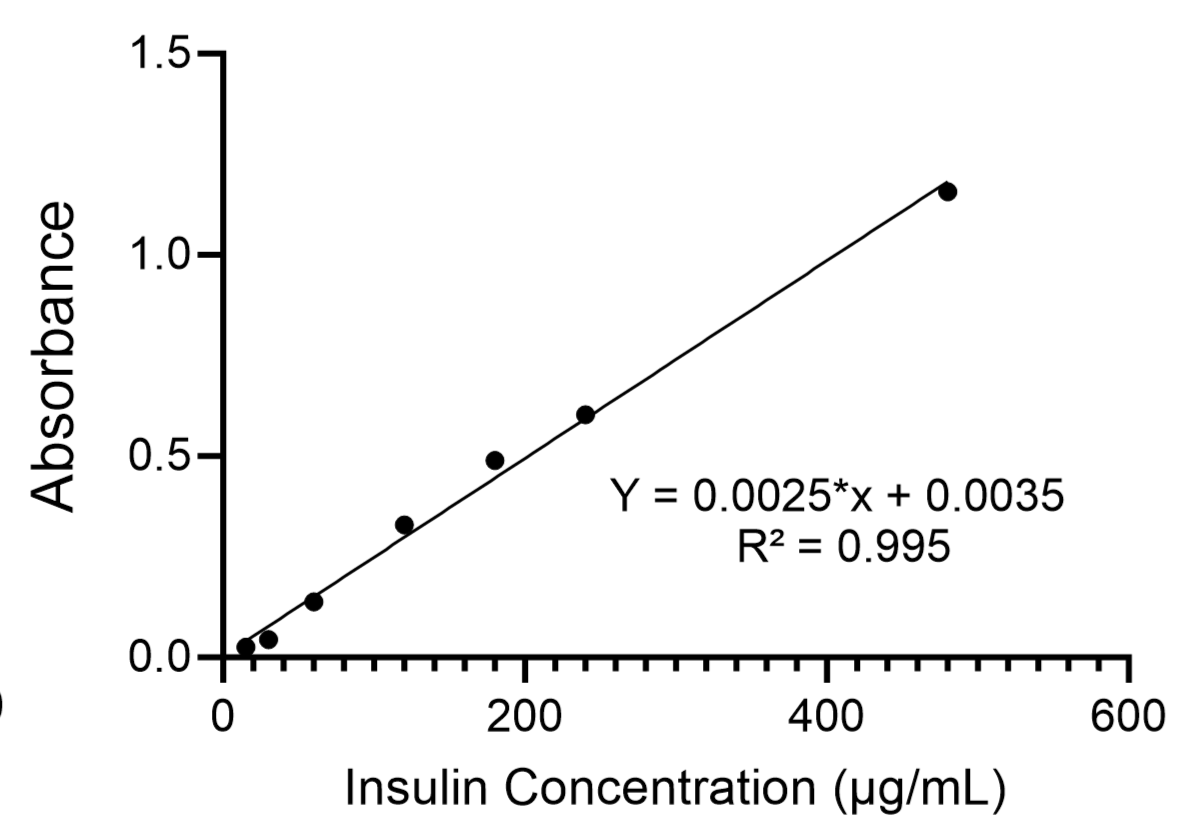


**Figure S1.** Standard curve for insulin concentration (µg/mL) vs absorbance (Abs).


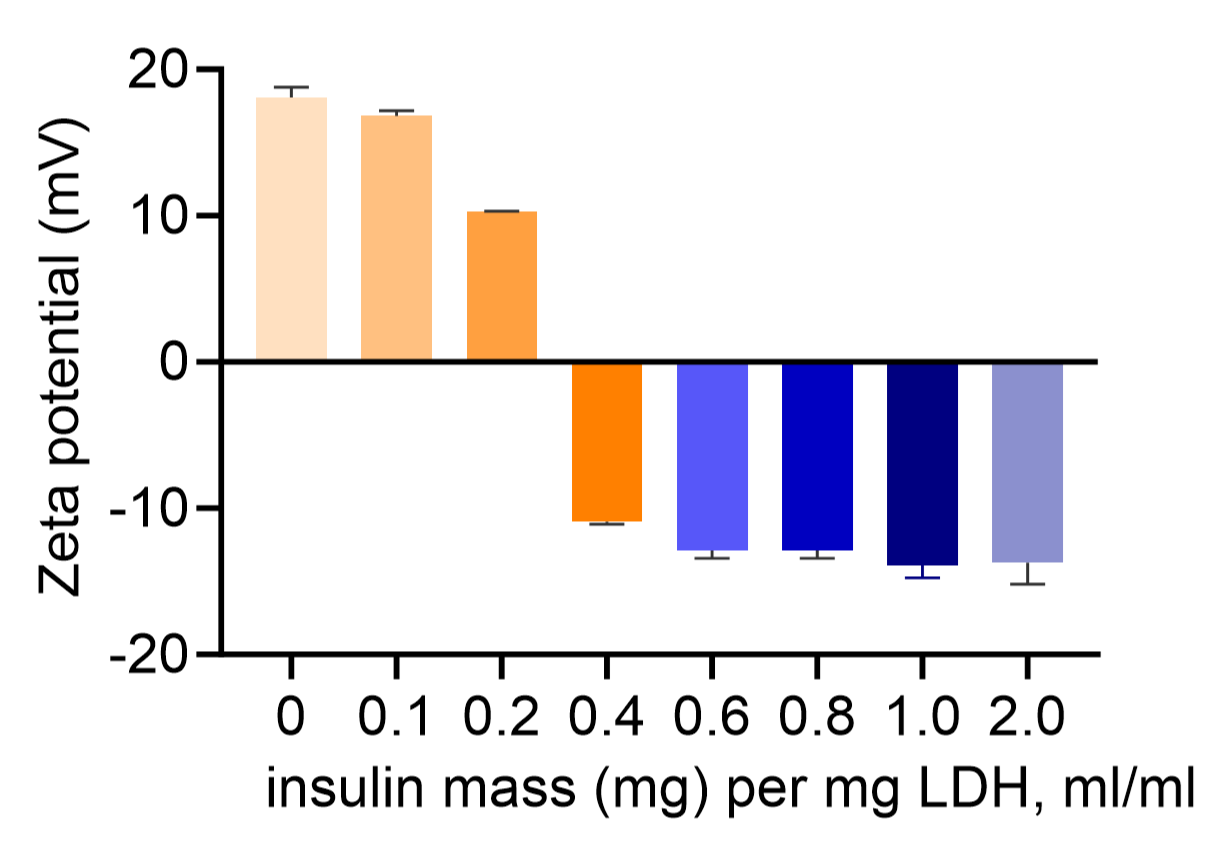


**Figure S2.** The zeta potentials of different LDH@insulin formations. 0.1-2.0 means during formulation, 0.1-2.0 mg/mL insulin was added into 1 mg/mL LDH nanoparticles.


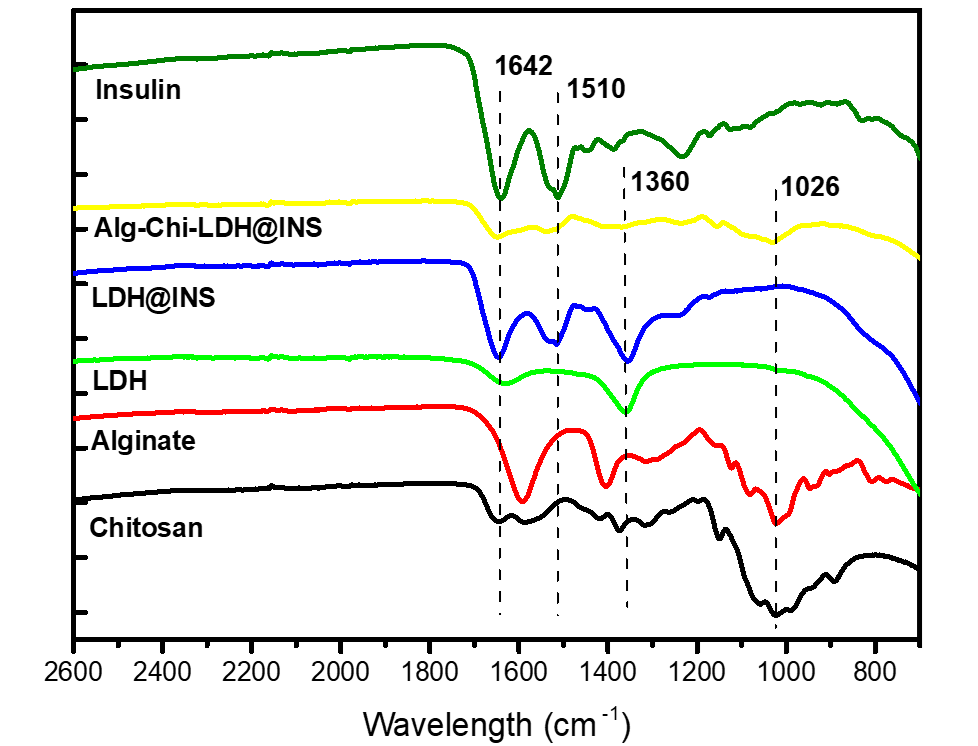


**Figure S3.** Fourier-transform infrared spectroscopy (FTIR) of chitosan, alginate, LDH, LDH@INS, Alg-Chi-LDH@INS, and insulin (INS).


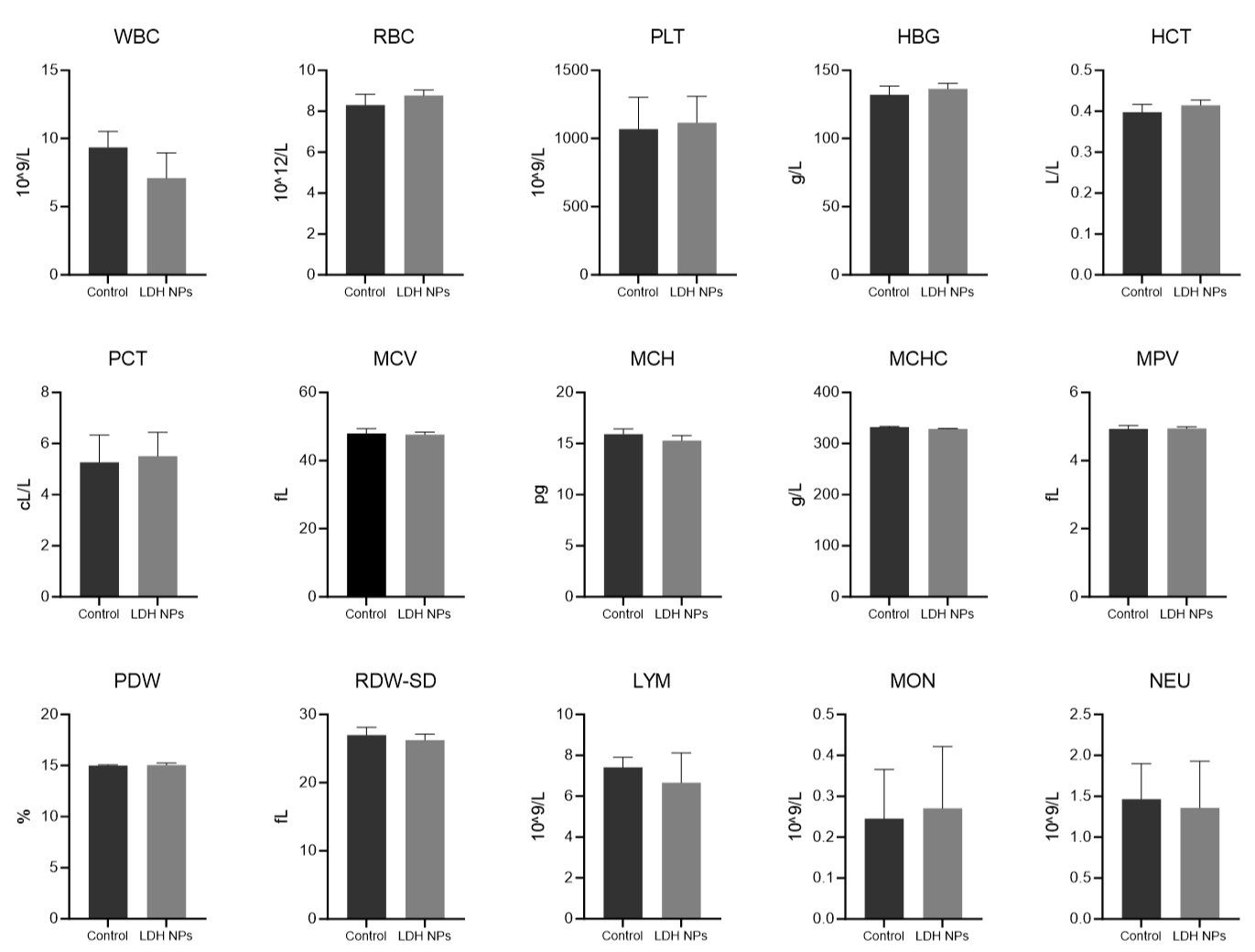


**Figure S4.** Haematological parameters of mice (n=5) treated with PBS solution as control and insulin-free LDH nanoparticles (NPs). There are no significant changes in various parameters as observed between mice with oral administration of PBS or LDH nanoparticles. Various parameters are leukocyte parameters (white blood cells (WBC), percentage of lymphocytes (%LYM), percentage of monocytes (%MON), granulocytes (GRA)); erythrocyte parameters (red blood cell (RBC), haemoglobin (HGB), haematocrit (HCT), mean corpuscular volume (MCV), mean corpuscular haemoglobin (MCH), mean corpuscular haemoglobin concentration (MCHC), red blood cells distribution width-standard deviation (RDW-SD)); thrombocyte parameters (platelet (PLT), mean platelet volume (MPV), platelet distribution width (PDW), neutrophils (NEU)).


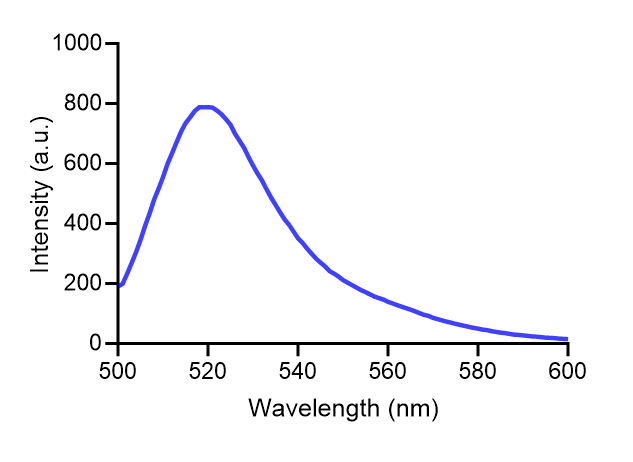


**Figure S5.** The fluorescence emission spectrum of FITC-conjugated insulin in aqueous solution at the excitation wavelength of 495 nm. FITC, fluorescein isothiocyanate.
